# Autism Associated With Anti-NMDAR Encephalitis: Glutamate-Related Therapy

**DOI:** 10.3389/fpsyt.2019.00440

**Published:** 2019-06-21

**Authors:** Ruu-Fen Tzang, Chuan-Hsin Chang, Yue-Cune Chang, Hsien-Yuan Lane

**Affiliations:** ^1^Department of Psychiatry, Mackay Memorial Hospital, Taipei, Taiwan; ^2^Mackay Medicine, Nursing and Management College, Taipei, Taiwan; ^3^Agricultural Biotechnology Research Center, Academia Sinica, Taipei, Taiwan; ^4^Department of Mathematics, Tamkang University, Taipei, Taiwan; ^5^Department of Psychiatry, China Medical University Hospital, Taichung, Taiwan; ^6^Graduate Institute of Biomedical Sciences, China Medical University, Taichung, Taiwan; ^7^Department of Psychology, College of Medical and Health Sciences, Asia University, Taichung, Taiwan

**Keywords:** autoimmune autism, glutamate-related therapy, anti-N-methyl-D-aspartate receptor encephalitis, immune therapy, dysfunctional glutamate neurotransmission

## Abstract

The purpose of this review is to correlate autism with autoimmune dysfunction in the absence of an explanation for the etiology of autism spectrum disorder. The anti-N-methyl-D-aspartate receptor (anti-NMDAR) autoantibody is a typical synaptic protein that can bind to synaptic NMDA glutamate receptors, leading to dysfunctional glutamate neurotransmission in the brain that manifests as psychiatric symptoms (psychosis, hallucinations, and personality changes). Detection of autoantibodies, cytokines, decreased lymphocytes, serum immunoglobulin level imbalance, T-cell mediated immune profile, maternal infection history, and children’s infection history can all be vital biological markers of autoimmune autism. Diagnosing autoimmune encephalitis sooner can increase the effectiveness of curative treatments—such as immune therapy or immune modulatory therapy—that may prevent the long-term consequence of being misdiagnosed with autism spectrum disorder. Glutamate therapy primarily normalizes glutamate neurotransmission and can be a new add-on intervention alongside antipsychotics for treating autoimmune autism.

## Introduction

Autism spectrum disorder (ASD) is characterized by symptoms of impaired communication, stereotyped behavior, difficulty with social interaction, and certain repetitive or unusual behaviors. The current prevalence of ASD is estimated to be 1.5% or higher in developed countries (1); however, the etiology of autism remains largely unexplained. At the beginning of the 1980s, researchers began to apply the “immune hypothesis of schizophrenia” to explain immune-dysfunction–induced neuroinflammation as a cause for the symptoms of schizophrenia (2). Since autism has some overlapping features with schizophrenia, for example both have disturbed cognitive and social function, and there are neurobiological (brain volumes) and genetic (e.g., involvement of the same genes or chromosomal locations) domains both in autism and schizophrenia (3). Therefore, researchers also apply “neuroinflammation hypothesis of ASD” to regard autism as a disorder of autoimmune dysfunction (4, 5) or as autoimmune autism.

At least 69% of individuals with a diagnosis of ASD have been known to have neuroinflammation or encephalitis (6). Specifically, the so-called “anti-brain autoantibody” may damage fetal or children’s brain cells, eventually leading to children falling into an autistic or regressive state. Such brain-reactive antibodies causing autistic symptoms may elucidate the exploration of autism’s etiology and suggest practical anti-inflammatory management protocols for children with ASD (7). Thus, this review introduces the potential pathogenicity of autism, which could explain why autoimmune dysfunction leads to autistic symptoms (indicated herein as “autoimmune autism”).

Furthermore, we emphasize that psychiatrists should more quickly recognize autoimmune encephalitis—anti-N-methyl-D-aspartate receptor (anti-NMDAR) encephalitis in children and adolescent patients and treat them as soon as possible, because timely immune therapy or glutamate therapy is beneficial for children with autism. In addition, through a literature review, we review four hypothetical pathways that correlate autism with dysfunctional autoimmunity.

## Dysfunctional Autoimmunity-Origin from Mother

On the basis of evidence that some of children with autism have mothers with rheumatoid arthritis, celiac disease, or a family history of type 1 diabetes (8), the symptoms of autism can be explained using the following exposure model. The fetus is exposed to prenatal antibodies from the dysfunctional maternal familial autoimmune system. The maternal pathogenic IgG antibodies or “induced maternal autoantibody-related ASD antibodies” cross the placenta and interact with the receptors in the fetal brain, eventually resulting in disruption of the brain cells (9–11).

An infection in the mother or child can trigger more brain damage in the fetus. If a mother with possible maternal familial autoimmune dysfunction develops an infection during pregnancy, this infection can trigger the autoimmune dysfunction to become severe, and maternal prenatal antibodies will then start attacking the brain of the fetus, leading to symptoms of autism (8); this is called “maternal-infection–triggered immune dysfunction.” Children may develop the social, functional, and behavioral deficits observed in ASD owing to impaired neurodevelopment triggered by infection (5). Therefore, history of maternal immune dysfunctional disease or maternal infection is vital for diagnosing autoimmune autism.

## Dysfunctional Autoimmunity-Origin from Child

Laing et al. (1989) demonstrated that when rabbits were inoculated with influenza A virus, the induced antibodies cross-reacted with a brain-specific protein found in the hippocampus, cortex, and cerebellum of the rabbits and caused brain cell damage. Various viral infections or immunizations during a child’s growth period—such as encephalitis associated with potassium channel complex antibody, NMDAR encephalitis, and Hashimoto’s encephalitis or vaccination induced anti-NMDAR encephalitis (12, 13)—all may cause several types of autoimmune encephalitis (5).

The anti-NMDAR autoantibody is a typical synaptic protein found in the nerve cells of teratomas. This autoantibody can bind with synaptic NMDA glutamate receptors in the brain, thereby causing dysfunctional glutamate neurotransmission and leading to psychiatric symptoms, such as psychosis, hallucinations, and personality changes (14). Recently, anti-NMDAR encephalitis has been frequently noticed after viral infection rather than originating from teratomas. The virally induced anti-NMDAR autoantibody may also attack the NMDA receptors of brain cells, causing hypofunction of glutamate neurotransmission in children with autism.

Such anti-NMDAR encephalitis is a type of reversible neuron dysfunction caused by autoimmune dysfunctional autoantibodies against NMDAR (15). Several cases of anti-NMDAR encephalitis have indeed been reported in patients with autism (16–18). Children with anti-NMDAR encephalitis symptoms that mimicked autistic regression recovered after timely immune therapy (19–21).

Approximately 30%–70% of autistic patients have circulating anti-brain autoantibodies (4). The autoantibodies in autistic patients—including anticardiolipin, β2-Glycoprotein 1, and antiphosphoserine antibodies (21); anti-double-stranded DNA antibodies (22); and antinucleosome-specific antibodies (23)—all act as anti-brain autoantibodies and play roles in disrupting neurotransmission by attacking specific neurotransmitter receptors. More specific autoantibody detection tools should be developed clinically to help autistic patients receive an accurate diagnosis instead of being misdiagnosed with autism.

## Autoimmune Autism After Interface Theories

Not all children develop autoimmune dysfunction after a viral infection. Through the “Immune-Mediated Two-Hit Model for Psychosis” theory, it can be understood that only the brains of a specific group of children will be attacked. These children possibly have mothers with activated monocytes or microglia caused by maternal stress and inflammation paradigms during a prior pregnancy. When inflammation or some viral infection hits the memorized immune system or activating microglia of children, these children develop severe autoimmune abnormalities, and the second hit blows their neuronal circuitry (24). Here, prior maternal stress and inflammation induce a memorized immune system or activate microglia in children’s brains, leading to the development of autistic symptoms in children during infections that occur in the early neurodevelopmental period.

## Autoimmune Autism—Cytokine Level

Cytokines [including chemokines, interferons, interleukins (ILs), lymphokines, and tumor necrosis factor (TNF)] are small proteins used in cell signaling. Cytokine deregulation occurs after overactivation of the immune system because of infection, injury, or inflammation (4). Elevated serum levels of IL-6 and IL-17A (25), increased serum IL-6 level (26), and increased expression of the inflammatory molecules IL-1β, IL-6, IL-17, and TNF (27) have been observed in children with autism.

Proinflammatory cytokines promote inflammation and have also been found to be highly increased in the peripheral blood of patients with an ASD diagnosis in comparison with controls (28, 29). Furthermore, the proinflammatory cytokines TNF-alpha, IL-6, granulocyte-macrophage colony-stimulating factor, Th1 cytokine (interferon-gamma), and chemokine (IL-8) are increased in the brains of individuals with ASD (30).

Most crucially, an increased level of one type of cytokine (IL-6) may alter the tryptophan/kynurenine pathway, which is closely related to glutamatergic neurotransmission (31). Assessing the aforementioned cytokine markers would allow researchers to diagnose autoimmune encephalitis accurately instead of misdiagnosing patients with autism. For children with autoimmune ASD symptom, the role of cytokines in ASD symptoms is that an autistic child may have autoimmune encephalitis induced glutamate hypofunction in his brain. Therefore, glutamate therapy could be used as the effective therapeutic strategy in restoring glutamate receptor’s function (32) and cytokine alterations. However, no study is available on using cytokine as the biomarkers of immune function to assess the effect of glutamate therapy in either ASD or schizophrenia. That will be an interesting study in future.

## Autoimmune Autism—T-Cell-Mediated Autoimmunity

In addition to an imbalance of cytokine levels in the serum or cerebrospinal fluid, signs of T-cell activation are reliable indicators of autoimmune dysfunction in autism. T-cell-mediated autoimmunity participates in the functions of physiological defense, maintenance, and brain repair. Autoimmune T-cell deficiency or T-cell imbalance alters the activation profile of T-cells (33)—the innate proinflammatory response—with increased T-cell activation or a skewed display (34). Moreover, it significantly increases the T helper cell 1 and 2 (Th1/Th2) ratio (30). All these actions are associated with autoimmune autism.

In summary, in *ex vivo* studies, immune cells including abnormal or skewed T helper cells (Th1 and Th2), cytokine profiles, decreased lymphocytes, decreased T-cell mitogen response, and an imbalance of serum immunoglobulin levels (35) have all been linked with autoimmune encephalitis and autistic symptoms.

## Autoimmune Autism—Nuclear Factor Kappa-Light-Chain-Enhancer of Activated B-Cells

Nuclear factor kappa-light-chain-enhancer of activated B-cells (NF-κB) is a protein found in almost all cell types. This protein mediates the regulation of cellular immune responses by promoting the expression of inflammatory cytokines and chemokines as well as by establishing a feedback mechanism that can produce chronic or excessive inflammation. Approximately 45% of a subgroup of children with autism have low natural killer (NK) cytotoxic cell activity (36). NF-κB is more aberrantly expressed in the orbitofrontal cortex of autistic patients than in controls. Specifically, the NF-κB of resident immune cells in brain regions are part of a molecular cascade indicating a more severe inflammation, which is associated with the behavioral and clinical symptoms of those with an ASD diagnosis (37).

## Autoimmune Autism—Immune-Related Genetic Polymorphism

Autism has been associated with autoimmune dysfunction and with immune-based genes including human leukocyte antigen (HLA)-DRB1 and complement C4 alleles. Such genes show aberrant immune activity during vulnerable and critical periods of neurodevelopment, participating in the generation of the neurological dysfunction characteristic of ASD (35). Higher expression of T-cell activation markers (HLA-DR, CD26) was noticed during a study of immune phenotyping of peripheral blood mononuclear cells in young autistic children but not in controls (38).

Additionally, patients with autism were discovered to have a significantly higher frequency of HLA-DRB1*11 allele than controls (39). This joint analysis of genotype and DNA methylation broadly demonstrates the potential of both brain and blood-based DNA methylation for insights into ASD and psychiatric phenotypes (40). The 16p11.2 mutations altered kynurenine pathway metabolism leading to abnormal glutamatergic activity in autism and may be the pathogenesis of ASD (31). Ghaleiha et al. suggested to use Memantine as an adjunctive treatment to restore NMDAR-dependent functionality before (41). Moreover, Memantine had a function targeting glutamate neurotransmission and already found to be the potential new and safe adjunctive treatment in children with ASD (42, 43).

## Autoimmune Autism—Diagnosis

Social cognitive impairment in children with autism originates from dysfunction in dopaminergic, serotonergic, noradrenergic, and glutamatergic neurotransmission in the brain after dysfunctional autoimmunity. Consequently, patients who develop autoimmune autism early in life may be misdiagnosed if their anti-NMDAR encephalitis or potential autoimmune-related disease remains unrecognized (44). Autoimmune dysfunctional autism requires immune therapy; therefore, earlier detection is essential to prevent a misdiagnosis of autism. Detection of autoantibody, cytokines, decreased lymphocytes, imbalance of serum immunoglobulin levels, and T-cell-mediated immune profile in addition to maternal infection history or children’s infection history can all be employed as biological markers of autoimmune autism.

## Autoimmune Autism—Treatment

Autoimmune dysfunctional autism requires immune therapy, which involves first-line immune therapy with pulse therapy in addition to intravenous immunoglobulin and plasmapheresis. Second-line immune therapy comprises rituximab or cyclophosphamide. In several cases, favorable treatment effects were reported after immune therapy if the child was noticed to have NMDAR-Ab in the serum and cerebrospinal fluid (16, 19–20, 21, 34, 45, 46). Recently, ASD drug development has focused on correcting synaptic dysfunctions; abnormalities in central oxytocin, vasopressin, and serotonin neurotransmissions, as well as neuroinflammation targets for new strategies to treat the core symptoms of ASD (47).

## Treatment: Second-Line Immune Therapy

Up to half of all patients treated for anti-NMDAR encephalitis reported poor treatment response and the failure of first-line immunotherapy (46). Among these patients with inadequate treatment response, approximately 65% showed substantial improvement after well-tolerated second-line immunotherapy (46, 48). Second-line therapy, most commonly rituximab and/or cyclophosphamide, is often required in patients without tumors and those with a delayed diagnosis (49). Rituximab directed against the CD20 antigen on the surface of B-lymphocytes leads to decreased maturation of B-cells into antibody-secreting cells and is a favorable immunotherapy option in anti-NMDAR encephalitis (50). Second-line immunotherapy using rituximab has been reported to have improved the long-term outcome in a 16-year-old female patient with autoimmune NMDAR encephalitis and autistic traits diagnosed earlier in childhood (20, 21). Therefore, this study suggests using second-line immunotherapy as soon as possible to help patients with autoimmune autism who have had a poor response to treatment after first-line immune therapy of pulse therapy, plasmapheresis, or immunoglobulins.

## Autoimmune Autism—Glutamate-Related Therapy

For patients with autoimmune autism who have responded poorly to immune therapy, have difficulty starting immune therapy, or are presently using antipsychotic treatment, immune-modulating therapy is the treatment of choice. Brain cells need glutamate (glutamic acid) to regulate the central nervous system. Accumulated neuroimaging, family, genetic and animal studies have demonstrated that glutamate (glutamic acid) can improve mood disturbance and executive function. In anti-NMDAR encephalitis, dysfunctional postsynaptic glutamatergic transmission in synapses leads to enhanced release of γ-Aminobutyric acid and hypofunction of glutamate secretion, thus the NMDAR agonist, e.g., D-cycloserine, sarcosine, and GLYX-13 (rapastinel) can modulate glutamatergic transmission (51). D-Cycloserine (DCS), a partial glycine B agonist at the NMDA receptor site, has been shown to improve sociability in patient with autism earlier (52) by normalizing glutamate neurotransmission. In recent years, D-cycloserine was also reported to be effective in improving stereotyped symptoms (53) and social reciprocity (54) in older adolescents and young adults with ASD.

Sodium benzoate, a D-amino acid oxidase inhibitor, was reported to be effective in children with communication difficulty with ASDs (55). Considering the challenge of earlier recognition of autoimmune autism, and the exorbitant cost of immune therapy in countries with poor medical knowledge of autoimmune autism, immune-modulating therapy or glutamate therapy can be a new add-on intervention alongside the current use of antipsychotics in treating autoimmune autism.

## Conclusion

Earlier diagnosis of autoimmune encephalitis increases the potential of curative treatments by enabling provision of timely immune therapy or immune modulatory therapy, which can prevent long-term consequences, such as being misdiagnosed with autism (16). An immunophenotyped patient with symptoms of autism may need to obtain a diagnosis of autoimmune encephalitis to avoid being misdiagnosed with ASD. Additionally, in children with the underlying syndrome presentation of childhood disintegrative disorder, early-onset schizophrenia, or late-onset autism (56); in children with first episode of psychosis (20); and especially when autistic symptoms follow a febrile illness (44), autoimmune autism including diagnosis of anti-NMDAR encephalitis or other autoimmune dysfunctional encephalitis should be considered as a possible organic cause.

## Author Contributions

R-FT, C-HC, and H-YL produced the idea to this review. R-FT, C-HC, Y-CC, and H-YL did the literature review. R-FT and C-HC made the draft of this paper. H-YL critically revised this manuscript. All authors read and approved the final manuscript.

## Conflict of Interest Statement

The authors declare that the research was conducted in the absence of any commercial or financial relationships that could be construed as a potential conflict of interest.
